# Extracellular Vesicles Isolated from the Brains of rTg4510 Mice Seed Tau Protein Aggregation in a Threshold-dependent Manner*

**DOI:** 10.1074/jbc.M115.709485

**Published:** 2016-03-30

**Authors:** Juan Carlos Polanco, Benjamin James Scicluna, Andrew Francis Hill, Jürgen Götz

**Affiliations:** From the ‡ Clem Jones Centre for Ageing Dementia Research, Queensland Brain Institute, University of Queensland, St. Lucia Campus (Brisbane), Queensland 4072, Australia,; the § Department of Biochemistry and Molecular Biology, Bio21 Molecular Science and Biotechnology Institute, University of Melbourne, Parkville, Victoria 3010, Australia, and; the ¶ Department of Biochemistry and Genetics, La Trobe Institute for Molecular Science, La Trobe University, Bundoora, Victoria 3086, Australia

**Keywords:** Alzheimer disease, exosome (vesicle), phosphorylation, Tau protein (Tau), tauopathy

## Abstract

The microtubule-associated protein tau has a critical role in Alzheimer disease and related tauopathies. There is accumulating evidence that tau aggregates spread and replicate in a prion-like manner, with the uptake of pathological tau seeds causing misfolding and aggregation of monomeric tau in recipient cells. Here we focused on small extracellular vesicles enriched for exosomes that were isolated from the brains of tau transgenic rTg4510 and control mice. We found that these extracellular vesicles contained tau, although the levels were significantly higher in transgenic mice that have a pronounced tau pathology. Tau in the vesicles was differentially phosphorylated, although to a lower degree than in the brain cells from which they were derived. Several phospho-epitopes (AT8, AT100, and AT180) thought to be critical for tau pathology were undetected in extracellular vesicles. Despite this, when assayed with FRET tau biosensor cells, extracellular vesicles derived from transgenic mice were capable of seeding tau aggregation in a threshold-dependent manner. We also observed that the dye used to label extracellular vesicle membranes was still present during nucleation and formation of tau inclusions, suggesting either a role for membranes in the seeding or in the process of degradation. Together, we clearly demonstrate that extracellular vesicles can transmit tau pathology. This indicates a role for extracellular vesicles in the transmission and spreading of tau pathology. The characteristics of tau in extracellular vesicles and the seeding threshold we identified may explain why tau pathology develops very slowly in neurodegenerative diseases such as Alzheimer disease.

## Introduction

Alzheimer disease (AD)^2^ is a debilitating, neurodegenerative disorder that is characterized by two prominent forms of fibrillar brain lesions: amyloid plaques composed of the peptide amyloid β (Aβ) and neurofibrillary tangles that contain hyperphosphorylated forms of the microtubule-associated protein tau (1). Aβ and tau are not merely signature molecules of AD, but they impair neuronal functions at a very early stage in disease, an observation that has spurred an interest in understanding what initiates the disease and how it propagates (2). Interestingly, tau pathology progresses through particularly well defined, stereotyped stages, starting in the locus coeruleus and slowly spreading via the entorhinal cortex and hippocampus toward the neocortex (3, 4). This pattern instigated several studies that support the concept that tau aggregates spread and replicate in what has been termed a prion-like manner, *i.e.* that the uptake of pathological forms of tau “seeds” causes the misfolding and aggregation of monomeric tau in recipient cells (5–7). This suggests that neuron-to-neuron transmission of tau seeds is a requirement for the spreading of tau pathology through the brain, a process that could potentially be achieved via various types of extracellular vesicles, tunneling nanotubes, uptake of free-floating tau aggregates and fibrils (8, 9), or by synaptically regulated mechanisms between interconnected neurons (10, 11). Although free tau aggregates have received considerable attention, whether extracellular vesicles that are physiologically released by mammalian cells have a role in tau propagation is slowly starting to be investigated in functional assays.

Extracellular vesicles (EVs) come in different sizes. Exosomes are defined as membranous extracellular nanovesicles (30–130 nm in size), whereas, traditionally, microvesicles are considered to fall within a size range of 100–1000 nm and apoptotic bodies within a range of 1000–5000 nm. Beyond their size discrimination, microvesicles and apoptotic bodies differ in their origin from exosomes. Microvesicles are cytoplasmic protrusions of the plasma membrane that are released in an outward process of budding or blebbing (12, 13). In contrast, exosomes are endocytic in origin and are formed by the inward budding of the endosomal membrane, which is progressively pinched off to generate and accumulate intraluminal nanovesicles. The late endosome, loaded with intraluminal nanovesicles, matures progressively into large multivesicular bodies. Multivesicular bodies may eventually fuse with the plasma membrane to release what are called exosomes into the extracellular space (12, 14). Interestingly, exosomes carry a range of proteins, mRNAs, and microRNAs. Not surprisingly, such cargos exert profound effects in recipient cells following cellular uptake. These vesicles are therefore considered important for intercellular communication and, in particular, the spreading of pathological agents from diseased cells, with important implications for cancer and, possibly, neurodegenerative diseases (14–16).

A putative role for exosomes in AD is supported by several observations. It has been reported that exosomes are associated with the Aβ peptide, the amyloid-precursor protein (APP) from which Aβ is derived, and additional products of APP processing (17–20). In addition, immunoelectron microscopy of AD brain tissue has revealed a physical association of exosome markers with neuritic Aβ plaques (17). Likewise, phosphorylated tau protein has been found associated with exosomes isolated from the blood and cerebrospinal fluid of AD patients (18, 21). However, despite the strong association between exosomes and phosphorylated tau, no functional assays have been performed to establish whether exosomal tau can seed the aggregation of endogenous tau and thereby contribute to tau pathology. Furthermore, larger extracellular vesicles such as microvesicles or ectosomes may also be involved in the spreading of tau pathology (22).

To clarify the pathological implications of exosome-associated Aβ, mouse models of AD have been instrumental to demonstrate that exosomes stimulate Aβ aggregation but also promote glia-mediated degradation of Aβ (20, 23). Furthermore, tau transgenic mouse models have linked exosomes to the function of microglia in the process of tau propagation (24). Taken together, these studies support the concept that reducing exosome secretion results in decreased plaque formation and also in reduced tau propagation. Therefore, pharmacological interventions to inhibit exosome release may offer a new treatment option for AD (23, 24). Here we sought to investigate whether EVs isolated from a tau transgenic mouse model carry tau seeds with the ability to induce tau aggregation in recipient cells and whether such tau in EVs is phosphorylated at epitopes found in AD patients (18, 21). We show that tau is indeed contained within EVs enriched for exosomes isolated from either wild-type mice or those with a pronounced tau pathology. We further demonstrate that the tau in these exosome-like EVs has specific characteristics and is able to seed the aggregation of endogenous tau in recipient cells in a threshold-dependent manner. Our data therefore suggest that exosome-like EVs carry tau seeds that could potentially contribute to the development of a tau pathology in AD.

## Experimental Procedures

### 

#### 

##### Mouse Strains and Collection of Brain Tissue

Animal experimentation was approved by the Animal Ethics Committee of the University of Queensland (approval nos. QBI/027/12/-NHMRC and QBI/412/14/NHMRC). For this study, we used female transgenic rTg4510 mice that express human four-repeat tau with the P301L mutation that is linked to hereditary tauopathy (25). Transgenic mice and gender-matched wild-type littermate controls were anesthetized and culled at 4–6 months of age. Following decapitation, brains were collected and transferred to a flask containing 5 ml of Hibernate-A (A12475-01, Life Technologies).

##### Isolation and Purification of Brain EVs

Exosome-like EVs were isolated from the interstitial space of the mouse brain using, with minor modifications, a protocol established previously (20). In brief, brains were dissected, and the cerebellum and olfactory bulbs were removed. These tissues were gently chopped before being incubated in 7 ml of 20 units/ml papain (LS003119, Worthington) in Hibernate-A for 20 min at 37 °C. The reaction was stopped with 14 ml of ice-cold Hibernate-A containing 1× Complete protease inhibitor mixture (Roche), 50 mm NaF, 200 nm Na_3_VO_4_, and 10 nm E-64 inhibitor (E3132, Sigma). The tissue was gently disrupted by pipetting with a 10-ml pipette, followed by a series of differential 4 °C centrifugations at 300 × *g* for 10 min, 2000 × *g* for 10 min, and 10,000 × *g* for 30 min to discard pellets containing cells, membranes, and nanodebris, respectively. The supernatant from the 10,000 × *g* centrifugation step was passed through a 0.22-μm syringe filter (Millex-GP, Millipore) and centrifuged at 100,000 × *g* for 70 min at 4 °C to pellet exosome-like EVs. The EV pellet was then resuspended in 25 ml of ice-cold PBS (17-516Q, Lonza), and the EV solution was centrifuged at 100,000 × *g* for 70 min at 4 °C. The washed EV pellet was resuspended in 2 ml of 0.95 m sucrose in 20 mm HEPES (15630-080, Life Technologies) and then inserted into a sucrose step gradient column (six 2-ml steps from bottom 2.0, 1.65, 1.3, 0.95, 0.6, and 0.25 m on top). The sucrose step gradient was centrifuged at 200,000 × *g* for 16 h at 4 °C. The original six 2-ml fractions were collected and resuspended in 6 ml of ice-cold PBS, followed by a 100,000 × *g* centrifugation for 70 min at 4 °C. Finally, the pellets were resuspended in 50 μl of PBS when EVs were used for cell assays or radioimmune precipitation assay buffer (150 mm NaCl, 50 mm Tris-HCl (pH 7.4), 0.25% (w/v) sodium deoxycholate, and 0.1% (v/v) Nonidet P-40) plus 1× Complete (Roche), 50 mm NaF, 200 nm Na_3_VO_4_, and 10 nm E-64 inhibitor when EVs were intended for Western blots. For Western blotting, EV lysates in radioimmune precipitation assay buffer were quantified for protein content with a microassay (DC protein assay, Bio-Rad). We also prepared cell lysates in radioimmune precipitation assay buffer using the cells from the 300 × *g* pellets obtained in the course of the EV isolations, which were used as positive controls for the Western blots.

##### Cell Culture and Tau Seed Transfections

We used modified HEK-293T cell lines provided by Dr. Marc Diamond. Two cell lines were polyclonal and stable for tau RD-CFP and tau RD-YFP, respectively. The third cell line was the FRET tau biosensor cell line that is monoclonal and expresses both tau RD-CFP and tau RD-YFP (26). The cell lines were grown in DMEM (Life Technologies) supplemented with 10% fetal bovine serum (12003C, Sigma), 100 units/ml of penicillin (Life Technologies), 100 μg/ml of streptomycin (Life Technologies), and 2 mm GlutaMAX (Life Technologies) at 5% CO_2_ and 37 °C in humidified air. Cells were seeded at 1.5 × 10^5^ cells/well in 12-well plates (Corning) 24 h before treatments. Liposome-mediated transduction of tau seeds was performed by combining 10 μl of Lipofectamine 2000 (Life Technologies) in 100 μl of Opti-MEM (Life Technologies) with 100 μl of Opti-MEM containing “tau seeds” (usually 20 μg of protein for mouse brain-derived EVs or brain lysates and 5 μg of protein equivalents for HEK293-derived EVs), mixing and incubating for 20 min at room temperature and then adding complexes dropwise to the cells. Cells were incubated with transfection complexes for 24 h. Tau seeds in the form of PBS brain cell lysates were prepared by making a cell suspension with the cells from the 300 × *g* pellet obtained during EV isolations in PBS (plus 1× Complete (Roche), 25 mm NaF, and 200 nm Na_3_VO_4_), extracting the proteins by sonication (six times for 10 s at 50% amplitude, Sonics VCX130) and clearing the PBS cell lysates by centrifugation (20,000 × *g* for 10 min).

##### Isolation and Purification of HEK293-derived EVs

Exosome-like EVs were isolated from the cell-conditioned medium (CCM) of HEK293 tau biosensor cells. Briefly, tau biosensor cells were transfected with either WT mouse brain lysates in PBS or transgenic (Tg) rTg4510 brain lysates using Lipofectamine, scaling up the culture to 10 T75 flasks per treatment as described above. After lysate treatments, the media of WT and Tg cell cultures were changed with exosome collection medium composed of DMEM (Life Technologies) supplemented with 5% exosome-depleted fetal bovine serum, 100 units/ml penicillin (Life Technologies), 100 μg/ml streptomycin (Life Technologies), and 2 mm GlutaMAX (Life Technologies). Exosome-depleted fetal bovine serum was prepared by centrifugation at 120,000 × *g* for 18 h, followed by filter sterilization of the supernatant. The CCM was collected every 24 h, and fresh exosome collection medium was added to the cells over 3 days. The CCM from each day was centrifuged at 2000 × *g* for 15 min, and then the supernatant was centrifuged at 10,000 × *g* for 30 min. An exosome-like EV pellet plus contaminating proteins were obtained by ultracentrifugation at 120,000 × *g* for 70 min. EV pellets from CCM were pooled after 3 days, washed with 25 ml of PBS, and again ultracentrifuged. The pooled EV pellet was resuspended in 2 ml of 0.95 m sucrose in 20 mm HEPES (15630-080, Life Technologies) and then purified on a sucrose step gradient column as described above for mouse brain-derived exosome-like EVs. After ultracentrifugation of the sucrose gradient, only fraction 3 (0.95 m sucrose) was recovered for further analysis.

##### Western Blotting Analysis

Protein quantification of exosome-like EV lysates showed that the sucrose gradient fraction 3 (F3 = 0.95 m sucrose) contained the highest protein concentration of all fractions. For brain-derived EVs, we used a volume of the F3-EV lysate containing 20 μg of protein and equivalent volumes for the remaining sucrose fractions for separation by 7–10% SDS-PAGE electrophoresis. For EVs obtained from HEK293 tau biosensor cells, we used 5 μg of protein equivalents of EVs. As positive controls for brain-derived EVs, we loaded 20 μg of wild-type and rTg4510 transgenic cell lysates on all gels run for Western blotting analysis. Whole cell lysates for Western blots were prepared with the 300 × *g* pellet, obtained during EV isolations, using a Dounce homogenizer and radioimmune precipitation assay buffer (plus 1× Complete (Roche), 50 mm NaF, 200 nm Na_3_VO_4_, and 10 nm E-64 inhibitor). As positive controls for HEK293-derived EVs, we loaded 5 μg of HEK293 tau biosensor cell lysates that had been prepared as above but disrupted the cells by sonication. Electrophoresed proteins were transferred onto immunoblot low-fluorescence PVDF membranes (170-4275, Bio-Rad) using the Trans-Blot Turbo transfer system (Bio-Rad). Membranes were blocked in Odyssey blocking buffer (Li-Cor) for 1 h at room temperature and then incubated overnight at 4 °C in primary antibodies prepared in a 1:1 mixture of Odyssey blocking buffer and Tris-buffered saline/0.1% Tween 20 (TBST). Membranes were washed with TBST three times for 10 min at room temperature, followed by a 1-h incubation with IRDye secondary antibodies (Li-Cor) diluted 1:10,000 in the same buffer as used for the primary antibodies. Finally, membranes were again washed three times in TBST, and the fluorescent signals were recorded using an Odyssey Fc imaging system (Li-Cor). Analysis and protein quantifications were performed using Image Studio software (Li-Cor). The following antibodies were used: anti-total tau Tau-5 (1:1000, MAB361, Millipore); anti tau RD4 (1:1000, 05-804, Millipore); and the anti-tau phospho-epitopes AT8 (1:1000, MN1020, Thermo Scientific), AT100 (1:1000, MN1060, Thermo Scientific), AT180 (1:1000, MN1040, Thermo Scientific), AT270 (1:1000, MN1050, Thermo Scientific), pS262 (1:1000, PA1-14422, Thermo Scientific), and Ser(P)-422 (1:1000, GTX86147, GeneTex). We also used the anti-exosome markers AIP1/Alix (1:1000, ABC40, Millipore) and flotillin-1 (1:500, sc-74566, Santa Cruz Biotechnology), the anti-neuronal marker TUJ1 (1:2000, MRB-435P, Covance), the anti-endoplasmic reticulum membrane marker calnexin (1:2500, ab22595, Abcam), the anti-mitochondrion marker VDAC1 (1:1000, ab15895, Abcam), the anti-early endosome marker EEA1 (1:1000, 07-1820, Millipore), and the normalizer anti-GAPDH (1:2000, ABS16, Millipore).

##### Proteolytic Surface Protein Shaving Assays

Exosome-like EVs from sucrose F3 (0.95 M sucrose) were diluted with PBS to a final volume of 40 μl at 1 mg/ml. Prelysed control samples were prepared by adding 2× lysis buffer (300 mm NaCl, 100 mm Tris (pH 7.4), 2% Triton X-100, and 2% sodium deoxycholate) and incubated on ice for 5 min, followed by sonication for 5 min in a sonicating water bath (SoniClean 80TD), after which they were cooled to 8 °C with ice. PK-positive samples (PK) (10 mm Tris and 1 mm CaCl (pH 8.5), Astral Scientific, AM0706) were prepared by adding PK to a final concentration of 100 μg/ml. A control without PK was included. The PK-containing samples (PK+) were incubated at 37 °C for 1.5 h. All unlysed samples were then centrifuged for 1 h at 16,100 × *g* at 4 °C, collecting the supernatant and pellets. The supernatants and lysed samples were precipitated by adding 160 μl (4× volume) of ice-cold methanol (precooled at −80 °C). The samples were then placed at −80 °C for 1 h before centrifugation at 16,100 × *g* at 4 °C for 1 h. The supernatants were discarded and the pellets left to air-dry for 15 min. Pellets from all samples were resuspended in 20 μl of SDS loading buffer + 5% β-mercaptoethanol and heated to 95 °C for 10 min prior to loading on 7–10% SDS-PAGE.

##### Electron Microscopy

Pellets from sucrose fractions resuspended in PBS were fixed in 1% glutaraldehyde for 30 min at room temperature. Fixed samples were loaded (6 μl) on 200 mesh carbon-coated formvar copper grids (GSAU200F-50, ProSciTech), washed twice with Milli-Q water, and negatively stained with 1.5% uranyl acetate. Electron micrographs were captured with a BM-UltraScan camera (Gatan) attached to an FEI Tecnai F30 electron microscope (Bio21 Molecular Science and Biotechnology Institute, University of Melbourne) operating at a 200 kV acceleration voltage and a JEOL 1010 transmission electron microscope (Center for Microscopy and Microanalysis, University of Queensland).

##### Determination of the Size Distribution and Concentration of EVs

Tunable resistive pulse sensing was conducted using a qNano instrument (Izon) to determine the diameter of the isolated vesicles or particles in solution. More specifically, samples were resuspended in PBS + 0.025% Tween 20 prior to measurement to reduce aggregation. EVs were driven through a qNano size-tunable nanopore (NP100, Izon) and detected one at a time as a transient change in the ionic current flow, which was denoted as a blockade event, with its amplitude representing the blockade magnitude. Because the blockade magnitude is proportional to the particle size, accurate particle sizing was achieved after calibration with particles of a known size (CPC100, Izon) using identical settings. Data processing and analysis were carried out using Izon Control Suite software v3.0 (Izon).

##### FRET Flow Cytometry

FRET flow cytometry was performed with RD-CFP, RD-YFP, RD-CFP + RD-YFP, and HEK-293T cell lines as described previously (26). In brief, cells were harvested with 0.05% trypsin-EDTA (Life Technologies), post-fixed in 2% paraformaldehyde (Sigma) for 10 min, and then resuspended in Hanks' balanced salt solution (14175095, Life Technologies) containing 1% fetal bovine serum and 1 mm EDTA. We used a BD FACSVantage SE DiVa cell sorter for the FRET analysis. To measure CFP and FRET, cells were excited using an I-50 laser (Coherent Inc.) tuned at 457 nm, and the emitted fluorescence was captured with a 485/22-nm and 585/42-nm filter, respectively. To measure YFP, cells were excited with an I-305 laser (Coherent Inc.) tuned to 514 nm, and the emitted fluorescence was captured with a 530/30-nm filter. To quantify FRET, we used a gating strategy similar to one described previously (26). For each experiment, either 20,000 or 40,000 cells/replicate were analyzed. Data analysis was performed using the FlowJo vX software (Tree Star). For some experiments, for measuring the uptake of protein aggregates and EVs labeled with far-red fluorescent tags, the red fluorescence was excited by a Spectra Physics model 127 HeNe laser emitting at 633 nm and collected through a 695/40 band pass filter.

##### Fluorescent Labeling of Proteins and Membranes

For a subset of experiments, cell lysates were fluorescently labeled on the N terminus of proteins and aggregates using an Alexa Fluor 647 protein labeling kit (A20173, Molecular Probes) following the instructions of the manufacturer. In the case of EVs, we labeled their membranes with the Cell Vue Claret far-red fluorescence cell kit (Sigma), which stably incorporates a far-red fluorescent dye (655/675 nm) with long aliphatic tails into the EV membrane. Exosome-like EV pellets from sucrose fraction 3 were resuspended in 1 ml of Diluent-C. Separately, 1 ml of Diluent-C was mixed with 6 μl of Cell Vue Claret. The EV suspension was mixed with the fluorescent dye solution, and the reaction was incubated for 4 min at room temperature. 6 ml of 1% bovine serum albumin was then added, and the mixture was ultracentrifuged at 100,000 × *g* for 70 min, washed with PBS, and ultra-centrifuged again. Finally, EVs were resuspended in PBS for further use. An outline of our strategies for labeling cell lysates and exosome-like EVs is provided in Fig. 6.

##### Confocal Microscopy of Cells Grown on Coverslips

Tau biosensor cells were grown on 18-mm coverslips with a poly-d-lysine coating (NeuVitro), adding 7 × 10^4^ cells/well in 12-well plates (Corning) 24 h before treatments. Transduction of far-red-labeled cell lysates (Alexa Fluor 647) and EVs (Cell Vue Claret) were performed as described for other cell culture experiments. After 24-h treatments, the cell culture medium was aspirated, and cells were washed three times with 1 ml of PBS/well/5 min, gently shaking and aspirating PBS between washes. The cells were then fixed with 1 ml of 2% paraformaldehyde in PBS for 20 min at room temperature. The fixative was discarded by aspiration, and a PBS wash was performed. For a subset of the experiments, flotillin-1 and AIP1/Alix immunofluorescence was performed using standard methods that permeabilize the cells using 0.1% saponin and reacting the primary antibodies with DyLight 405 goat anti-mouse (1:500, 35500BID, Thermo) and Alexa Fluor 555 goat anti-rabbit antibodies (1:500, A-21429, Life Technologies). In another subset of experiments, cells were only stained for 5 min with DAPI (Molecular Probes, 0.5 μg/ml in PBS) to visualize nuclear DNA. Finally, cells were washed with PBS, and coverslips were mounted on Superfrost Plus slides (Menzel-Glaser) using Vectashield Antifade mounting medium (Vector). Fluorescence images at ×20, ×40, and ×100 magnification were obtained with a Zeiss LSM 510 Meta confocal microscope. For fluorescent particle quantifications of tau aggregates, four non-overlapping ×20 magnification fields were taken per sample and analyzed with Open Source ImageJ software (version 1.47b, Wayne Rasband, National Institutes of Health, Bethesda, MD). Acquisition parameters remained invariable for all images. The fluorescence signal was adjusted by image segmentation, applying a threshold to specifically detect tau inclusions on the green channel and nuclei (DAPI) on the blue channel. Images with particles to quantify were converted to grayscale. The area of each particle was determined per image on each channel using ImageJ particle analysis. Total areas of tau inclusions were summed up, as well as total areas of nuclei. The ratio of the area occupied by tau inclusions over the area occupied by nuclei was calculated per image. The tau aggregate/nuclei ratios from four images were averaged, and standard deviation was calculated per sample.

##### Immunofluorescence of Extracellular Vesicles on Coverslips

5–20 μg of protein equivalents of extracellular vesicles (either untreated or previously treated with Lipofectamine) was placed on 18-mm coverslips with a poly-d-lysine coating (NeuVitro) and incubated with 400 μl of DMEM. After overnight incubation in 5% CO_2_ and 37 °C, the medium was aspirated, and the coverslips were directly fixed with 1 ml of 2% paraformaldehyde in PBS for 15 min at room temperature and then washed with PBS. The coverslips were then blocked with 3% BSA in PBS, followed by standard immunofluorescence with the anti-exosome markers AIP1/Alix (1:200, ABC40, Millipore) and flotillin-1 (1:50, sc-74566, Santa Cruz Biotechnology), which were detected with Alexa Fluor 555 goat anti-mouse (1:500, A21424, Life Technologies) and Alexa Fluor 488 goat anti-rabbit antibodies (1:500, A11034, Life Technologies). Coverslips were mounted on Superfrost Plus slides (Menzel-Glaser) using Vectashield Antifade mounting medium (Vector). Fluorescence images were obtained at ×63 with a ×2–3 zoom using a Zeiss LSM 710 confocal microscope.

##### ThT Spectroscopic Assay

Changes in fluorescence intensity of thioflavin T (ThT) were used to quantify the presence of misfolded tau aggregates in disrupted EVs *in vitro*. HEK293-derived EVs were disrupted using sonication (three times for 10 s at 20% amplitude, Sonics VCX130), and then the EV protein or the corresponding buffer (5 μl) was plated in triplicates in a 96-well black plate with a clear bottom (Corning 3603), followed by addition of 100 μl of ThT in PBS (20 μg/ml) per well. After mixing and a short incubation, ThT fluorescence was recorded (λexcitation, 440 nm; λemission, 482 nm) using a BMG CLARIOstar® microplate reader.

##### Statistical Analysis

Differences in expression levels were evaluated for statistical significance according to *p* values gained from a one-way ANOVA with a 95% confidence interval, calculated with GraphPad Prism 6 for Windows (GraphPad Software Inc.).

## Results

### 

#### 

##### EVs Isolated from the Brains of Wild-type and Tau Transgenic rTg4510 Tau Mice Present with Similar Physical Properties

Phosphorylated tau has been found previously to associate with exosomes in the cerebrospinal fluid of AD patients, suggesting that these vesicles may mediate the trans-neuronal spreading of tau pathology (21). Given that no strong association has been demonstrated between phosphorylated tau and exosomes in cultured cells (27–30), we investigated whether transgenic mice with a robust neuronal tau pathology would release exosomes carrying phosphorylated tau.

**FIGURE 1.**
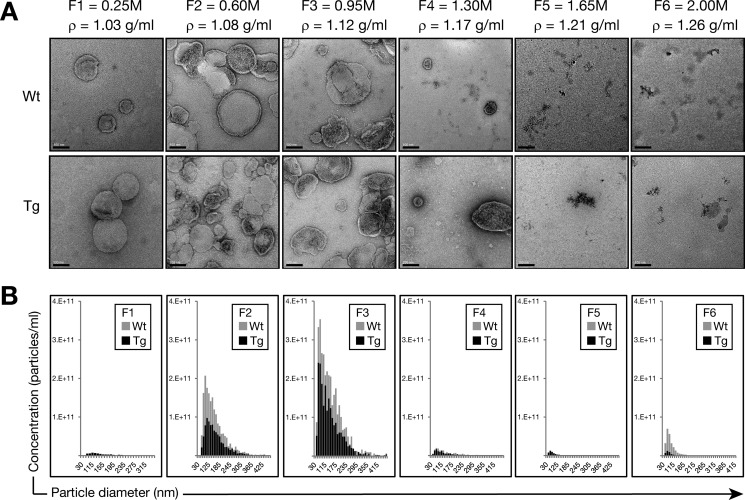
**Physical analysis of nanovesicles isolated from brains of P301L tau transgenic rTg4510 mice.** Exosome-like EVs were isolated from the brain extracellular space according to Perez-Gonzalez *et al.* (20). *A*, transmission electron microscopy analysis of sucrose F1-F6 shows nanovesicles mostly in F2 (0.6 m sucrose, ρ = 1.08g/ml) and F3 (0.95 m sucrose, ρ = 1.12g/ml). *Scale bars* = 100 nm. *B*, tunable resistive pulse sensing using a qNano instrument (Izon). Concentration histograms of size distributions (sizes in nanometers *versus* particles per milliliter) are shown for each population in the different sucrose fractions (F1-F6). In agreement with transmission electron microscopy, the highest concentrations of nanovesicles were found in F2 (0.6 m) and F3 (0.95 m). The size distribution is compatible with exosomes for both WT and rTg4510 Tg nanovesicles, with an average size of 130 nm in F3 (0.95 m sucrose) and the most common nanovesicle size of ∼74 nm for both WT and Tg samples. However, some larger extracellular vesicles (>130 nm) were co-isolated with these potential exosomes. More exosome-like EVs were obtained from the WT samples than the Tg samples.

To maximize the potential yield of tau-containing exosomes, we used a series of centrifugation steps to isolate EVs from the extracellular space of murine brains of P301L tau-expressing rTg4510 transgenic mice that present with a pronounced tau pathology at an early age (25). Following final purification on a sucrose gradient, we obtained six fractions that were analyzed by EM for purity and the presence of EVs and with a qNano instrument (Izon) via tunable resistive pulse sensing to determine their size distribution. We found that F3 (0.95 m sucrose, ρ = 1.12 g/ml) exhibited the highest concentration of nanovesicles (Fig. 1), with a size compatible with that of exosomes according to both EM analysis (Fig. 1*A*) and tunable resistive pulse sensing (Fig. 1*B*). However, we also detected larger extracellular vesicles (>130 nm) co-isolated with exosomes (Fig. 1, *A* and *B*). Therefore, we termed the nanovesicles in F3 “exosome-like EVs.”

Concentration histograms showed a higher concentration of EVs in WT samples (Fig. 1*B*), possibly reflecting the 17% higher mass of their brains compared with Tg mice (average weight: WT, 0.52 ± 0.01 g; Tg, 0.43 ± 0.01 g). However, it is also possible that endocytic trafficking in neurons transgenic for mutant tau is impaired, as evidenced by the accumulation of endosomes and exosomes, resulting in the release of less exosomes into the extracellular space of the brain (31).

##### Tau Protein Is Present in EVs Derived from Both Wild-type and Tau Transgenic Mice

**FIGURE 2.**
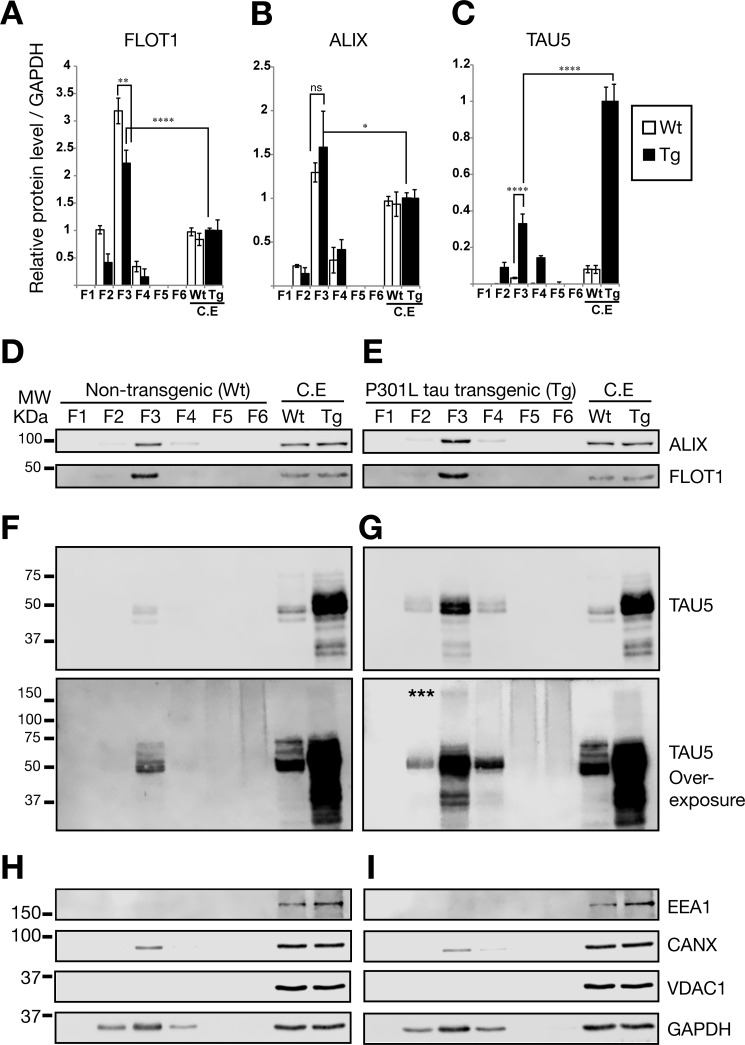
**Analysis of protein contents in isolated EVs.** Shown are quantitative Western blotting analysis of FLOT1, ALIX, and TAU expression. The same amount of protein (20 μg) was loaded for F3 EVs and whole control cell extracts (*C.E*). *Error bars* represent S.E. (*n* = 3). *, *p* < 0.05; **, *p* < 0.01; ****, *p* < 0.0001. *A*, quantification of protein levels for FLOT1, showing a very strong enrichment. *B*, protein levels for ALIX, an exosome marker of the endocytic pathway, were also enriched. *C*, quantification of protein levels for total tau. *D–I*, representative Western blots for different sucrose fractions of exosome isolations from WT and Tg mice, showing ALIX and FLOT1 expression (*D* and *E*) as well as total tau (mouse and human) detected with the Tau5 antibody (*F* and *G*). Note the *bottom panels* (*F* and *G*) for Tau5 overexposure, revealing high molecular weight (*MW*) proteins in F3, stronger around 150 kDa (*asterisks*), indicating potential trimers of tau in EVs. F5 and F6 show a continuous high molecular weight smear, indicating that some of the aggregates visualized by transmission electron microscopy (Fig. 1) could be free tau aggregates, which sediment at high concentrations of sucrose (59). *H* and *I*, detection of markers for cytoplasmic organelles in the sucrose fractions of exosome-like EVs: mitochondrial (VDAC1), early endosome (EEA1), and endoplasmic reticulum markers (calnexin) were tested.

We next confirmed, by protein quantification for the exosomal markers FLOT-1 and ALIX, that the extracellular vesicles we had detected indeed contained exosomes (Fig. 2). We found that levels of FLOT1 were strongly (∼3 times, *p* < 0.0001, ANOVA) enriched in sucrose F3 (0.95 m) compared with whole mouse brain lysates using the same amount of total protein (Fig. 2, *A*, *D*, and *E*). ALIX was also enriched in the F3 fraction, albeit at a lower level (∼1.5 times, *p* < 0.05, ANOVA) (Fig. 2, *B*, *D*, and *E*). Together, the enrichment of both FLOT1 and ALIX strongly supports the notion that the EVs observed in F3 indeed contained exosomes.

We next determined whether sucrose F3 from both wild-type and transgenic mice contains tau protein (Fig. 2, *C*, *F*, and *G*), indicating that this association is not triggered by or dependent on the presence of the transgene and that tau is physiologically secreted via EVs. Although we identified tau in both samples, the exosome-like EVs from the transgenic mice carried ∼10 times more tau protein than those isolated from wild-type mice (*p* < 0.0001, ANOVA) (Fig. 2*C*), an effect likely related to the higher levels of tau in rTg4510 mice. When protein equivalents were analyzed (20 μg), we also found that transgenic and wild-type EVs in F3 contained around 33% and 3.3%, respectively, of the tau observed in whole cell lysates normalized for protein levels (Fig. 2*C*). These results suggest that the tau protein content in exosome-like EVs tends to mirror that of the cytoplasm, revealing a ratio of wild-type *versus* transgenic tau of ∼1:12.5 (Fig. 2, *C*, *F*, and *G*). Furthermore, when the blot for total tau (Tau5) was overexposed (Fig. 2, *F* and *G*, *bottom panels*), we observed that only transgenic exosome-like EVs in F3 present high molecular weight proteins with a stronger tau signal around 150 kDa compatible with a trimer.

We also investigated whether internal cytoplasmic markers were absent in the sucrose fractions of the exosome-like EVs (Fig. 2, *H* and *I*). We did not detect mitochondrial (VDAC1) or early endosome proteins (EEA1; Fig. 2, *H* and *I*). We detected a minor presence of the endoplasmic reticulum marker calnexin (Fig. 2, *H* and *I*, *CANX*), which could indicate a minor contamination with internal or synaptic vesicles released during the dissociation of brain tissue. However, according to the exosome database, calnexin has been detected in a substantial number of exosome isolations from both biological fluids and supernatants of cultured cells (32, 33).

##### Tau Is Differentially Phosphorylated in Exosome-like EVs

**FIGURE 3.**
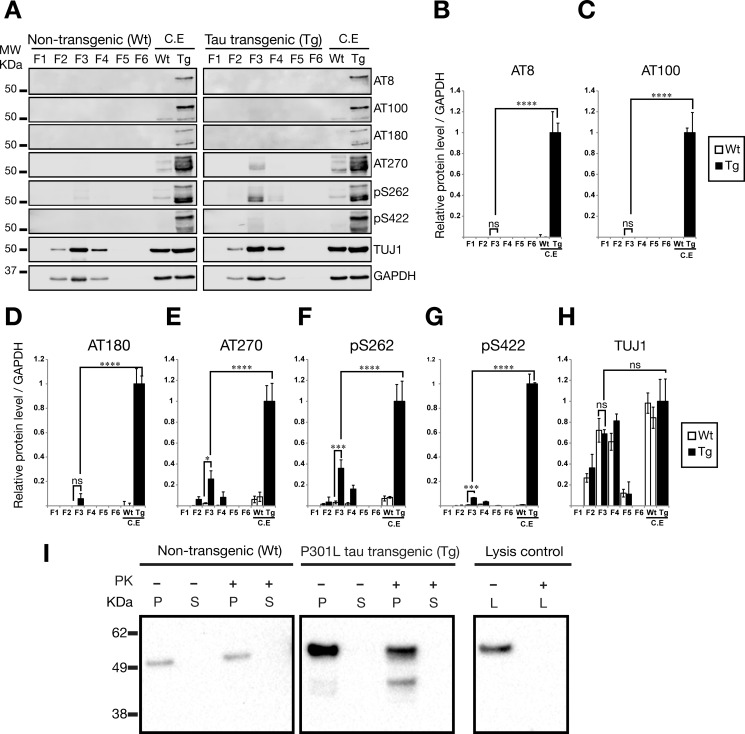
**Phosphorylation-dependent tau epitopes are present at very low levels in exosome-like EVs isolated from mouse brains.** Shown are Western blotting analysis of sucrose fractions F1-F6, testing different AD-associated phospho-tau epitopes. 20 μg of total protein was loaded for F3 exosome-like EVs and whole cell lysate controls. *Error bars* represent S.E. (*n* = 3). *, *p* < 0.05; ***, *p* < 0.001; ****, *p* < 0.0001; *ns*, not significant. *A*, representative Western blots for paired helical filament-tau phospho-epitopes and additional proteins. *MW*, molecular weight; *C.E*, cell extract. *B–G*, quantification of protein levels for (*B*) AT8, (*C*) AT100, (*D*) AT180, (*E*) AT270, (*F*) Ser(P)-262, and (*G*) Ser(P)-422. Analysis reveals that F3, enriched for exosome markers, contains the phospho-tau epitopes AT270, Ser(P)-262, and Ser(P)-422. Interestingly, the AT8, AT100, and AT180 phospho-epitopes are not detected in exosome-like EVs. *H*, protein levels for neuron-specific class III β-tubulin (TUJ1), demonstrating the neuronal origin of the EVs. *I*, PK surface shaving was performed with exosome-like EVs in sucrose fraction F3 (0.95 m sucrose). Total tau was assayed with the Tau5 antibody. No tau was identified in the supernatant (*S*) after PK treatment. Tau was associated with the pellet (*P*) of intact exosome-like EVs after centrifugation in both WT and Tg mice. Tau protein was completely degraded when PK was added to lysed EVs (*L*).

We next investigated whether the tau present in exosome-like EVs was phosphorylated at epitopes that are usually associated with AD and the formation of tau-containing paired helical filaments. We found that important paired helical filament-tau phospho-epitopes such as AT8 (Ser(P)-202/Thr-205), AT100 (Thr(P)-212/Ser-214), and AT180 (Thr(P)-231) were not detectable in exosome-like EVs from rTg4510 mice, whereas they were clearly detectable in whole cell lysates from the mice from which the EVs had been derived (Fig. 3, *A–D*). However, antibodies that recognize other fibrillar and pretangle tau structures, such as AT270 (Thr(P)-181), Ser(P)-262, and Ser(P)-422, were detected in exosome-like EVs isolated from rTg4510 mice (Fig. 3, *A* and *E–G*). Taken together, these results support the notion that tau phosphorylation in exosome-like EVs differs from that of total extracts in P301L tau transgenic rTg4510 mice, indicating that the absence, or comparatively much lower levels of AT8, AT100, and AT180, may have functional consequences for the seeding capability of tau originating from exosome-like EVs. To demonstrate the neuronal origin of the isolated EVs, we used an antibody for the neuron-specific class III β-tubulin (TUJ1), showing robust association of this marker with the brain-derived EVs (Fig. 3, *A* and *H*).

##### Tau Is Located inside Exosomes

There are several possibilities for how tau may be associated with exosomes. Such a protein could be physically located inside the lumen of exosomes (intravesicular), span the exosome membrane (transmembranous), or be associated with the outer side of the exosomal membrane without spanning the lipid bilayer completely. Previous reports of tau in exosomes have found tau inside the inner leaflet of exosomes isolated from PS19 mouse brains using immunoelectron microscopy of ultrathin sections (24). Furthermore, a biochemical method with increasing concentrations of sodium chloride also provided evidence of the location of tau inside extracellular vesicles (22). Here we investigated whether, in rTg4510-derived brain EVs, tau was also inside the vesicles. We used surface protein shaving assays in which the mouse brain exosome-like EVs were treated with PK to degrade proteins that were accessible on the surface. In a situation where tau is accessible from the surface, PK should partially or even completely degrade the protein, releasing fragments into the supernatant. However, we did not observe any tau fragments in the supernatants when we performed this assay or any strong reduction in total tau analyzed (Fig. 3*I*). Rather, tau was always found to be associated with the EV pellet, indicative of its protection by EV membranes, and it was only completely degraded by PK in the lysis control into minute fragments that appeared to have migrated out of the electrophoresis gel (Fig. 3*I*). Together, this demonstrates that tau is located inside exosome-like EVs from the brains of rTg4510 mice.

##### Exosome-like EVs Can Trigger the Aggregation of Endogenous Tau Protein in FRET Tau Biosensor Cells

It has recently been reported that FRET tau biosensor cells detect tau seeding activity with ultra-high sensitivity and specificity (26). These cells are an engineered HEK293T cell line that stably expresses the tau RD together with the pathological mutation P301S fused to either CFP or YFP (26). When both tau RD-CFP and tau RD-YFP are present, treatment with exogenous tau seeds leads to endogenous tau reporter protein aggregation, which generates a FRET signal that can be detected by flow cytometry or confocal microscopy.

**FIGURE 4.**
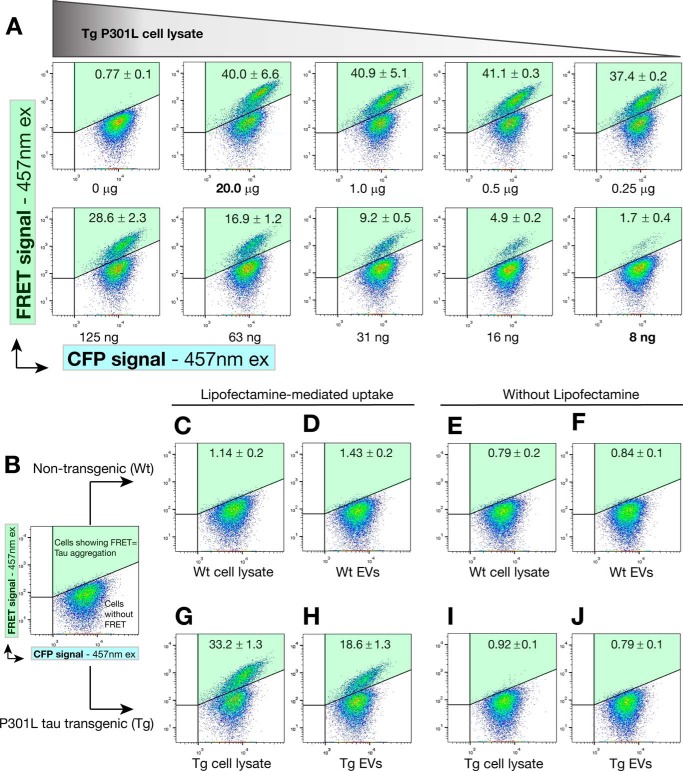
**Exosome-like EVs can induce endogenous tau aggregation in FRET tau biosensor cells.** FRET tau biosensor cells are HEK293T cells that express both tau-CFP and tau-YFP (RD domain). Cells were treated and analyzed by FRET flow cytometry after a 24-h treatment (*n* = 3, average ± S.D., 20,000 cells/experiment were analyzed). Representative flow cytometry plots show the FRET signal as detected in the right gate (Q2, shaded in *green*). *A*, titration of protein sample to determine concentrations near saturation of FRET tau biosensor cells. Cells were treated with decreasing amounts of rTg4510 transgenic brain cell lysates in the presence of Lipofectamine and analyzed by FRET flow cytometry after a 24-h treatment. 20 μg of protein in cell lysates is sufficient to generate a FRET signal that is not much lower than that obtained with only 1.0 or 0.5 μg of protein. Even with 8 ng of protein, a FRET signal is detected. Therefore, cells are saturated at 20 μg of cell lysate and detect tau seeds up to the low nanogram range. This implies that even minute amounts of tau seeds are detectable in 20 μg of protein. *ex*, excitation. *B–J*, analysis of the requirement of Lipofectamine. FRET tau biosensor cells were treated in the presence (*C*, *D*, *G*, and *H*) or absence (*E*, *F*, *I*, and *J*) of Lipofectamine, with vehicle (*B*) showing a lack of signal in the gate for FRET (upper right gate Q2, shaded *green*). *C–F*, none of the treatments with WT EVs or cell lysates can induce FRET. *G–J*, a strong FRET signal is detected with Tg cell lysates (*G*) and Tg EVs (*H*), but only in the presence of Lipofectamine. *I* and *J*, the same Tg samples without using Lipofectamine.

To investigate whether treatment with P301L tau-containing EVs can induce the aggregation of endogenous tau, we treated the biosensor cells with the exosome-like EVs for 24 h and measured the ensuing effects by FRET flow cytometry (Fig. 4). We first performed a titration assay to determine the amount of exogenous protein that would be close to saturation of the FRET cellular system in a 12-well format (Fig. 4*A*). Titrating the amounts of exogenous transgenic cell lysate along with Lipofectamine, we found that 20 μg of exogenous protein was more than sufficient to induce FRET events in a robust manner, although, in our hands, the FRET tau biosensor cells were able to detect tau seeds contained in up to 8 ng of P301L tau transgenic cell lysate (Fig. 4*A*).

Using vehicle (Fig. 4*B*) as well as non-transgenic (wild-type) exosome-like EVs and lysates as a control (Fig. 4, *C–F*), we subsequently tested 20 μg of total protein from transgenic exosome-like EVs and compared this with a 20-μg equivalent obtained from transgenic cell lysates (Fig. 4, *G–J*). We found that only EVs obtained from transgenic mice were able to generate a strong FRET signal (Fig. 4*H*). However, transgenic cell lysates showed almost twice the signal triggered by exosome-like EVs (Fig. 4*G*). In comparison, none of the non-transgenic samples induced a FRET signal (Fig. 4, *C–F*). We also noted that, for the incubation time chosen, P301L transgenic samples did not induce FRET in the absence of Lipofectamine (Fig. 4, *I* and *J*), a reagent reported to increase seed detection efficiency in the FRET tau biosensor system, although it is not considered necessary (26).

##### Longer Incubation with Seeds Induces Tau Aggregation without Lipofectamine

**FIGURE 5.**
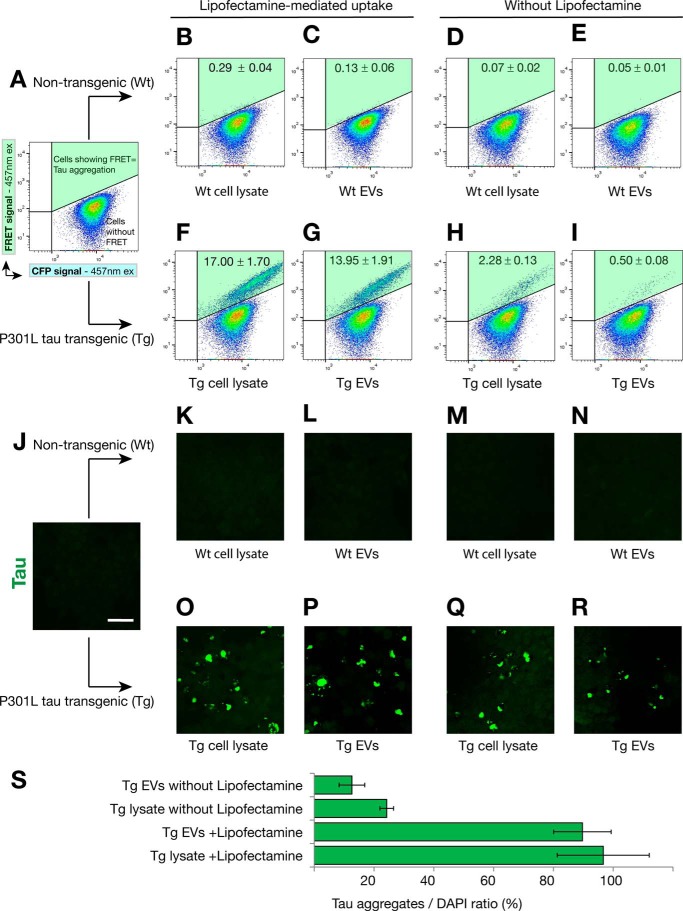
**Effect of an extended culture on the generation of the FRET signal and tau aggregation.** FRET tau biosensor cells were treated in the presence (*B*, *C*, *F*, and *G*) or absence (*D*, *E*, *H*, and *I*) of Lipofectamine and analyzed by FRET flow cytometry after a 72-h treatment (*n* = 3, average ± S.D.. 40,000 cells/experiment were analyzed). *A*, FRET tau biosensor cells treated only with Lipofectamine do not induce FRET (upper right gate Q2, shaded *green*). *ex*, excitation. *B–E*, none of the treatments with WT exosome-like EVs or cell lysates triggers FRET. *F* and *G*, a strong FRET signal is detected with Tg cell lysates (*F*) and Tg exosome-like EVs (*G*) in the presence of Lipofectamine. *H* and *I*, when Lipofectamine is omitted, a weak signal is detected for Tg lysates (*H*) and also for Tg EVs (*I). J–R*, parallel experiment using the same conditions was performed with cells grown on coverslips to then be analyzed by confocal microscopy. *Scale bar* = 50 μm. *O–R*, induced tau aggregates were visualized only with Tg samples. *S*, quantification of the ratio of the area occupied by tau inclusions normalized by the area occupied by nuclei 72-h after treatment (tau aggregates/DAPI in percent, *n* = 4).

We next sought to determine whether a longer incubation time would induce seeding of exosome-like EVs even in the absence of Lipofectamine (Fig. 5). Therefore, we performed the above assays with a 72-h incubation instead of a 24-h one. To avoid overconfluence after extended culture, the 72-h experiments were performed with three times fewer cells than used for the 24-h time points. Using vehicle (Fig. 5*A*) as well as non-transgenic EVs and lysates as a control (Fig. 5, *B–E*), we tested exosome-like EVs and transgenic cell lysates for the longer incubation times (Fig. 5, *F–I*). In this situation, we were able to detect a FRET signal of the transgenic lysates even in the absence of Lipofectamine treatment (Fig. 5, *H* and *I*), although the signal was ∼7-fold lower than with Lipofectamine (Fig. 5, *F* and *G*). A parallel experiment was performed using the same conditions but analysis by confocal microscopy (Fig. 5, *J–S*). We found that tau inclusions were only induced with transgenic samples (Fig. 5, *O–R*), and, in agreement with the FRET flow cytometry data, we indeed detected and quantified tau inclusions in the absence of Lipofectamine (Fig. 5, *Q–S*).

##### High Internalization of Tau Transgenic EVs and Protein Aggregates Induces Intracellular Tau Inclusions

We reasoned that the low seeding and FRET signal using flow cytometry in the absence of Lipofectamine could be due to three reasons. 1) It could be due to the existence of a threshold for the induction of endogenous tau aggregation. Accordingly, exosome-like EVs would seed with a lower efficiency than brain lysates because EVs carry ∼33% of the tau protein present in cells (Fig. 2*E*) and they also show lower levels of phosphorylated tau (Fig. 3). 2) It could be due to a difference in uptake, with either low or no uptake of exosome-like EVs compared with cell lysates. 3) It could be due to a difference in processing by the FRET tau biosensor cells given that EVs and cell lysates are different types of samples, *i.e.* lysates contain free tau aggregates, and EVs carry tau encapsulated by membranous vesicles (22, 24) (Fig. 3*I*).

**FIGURE 6.**
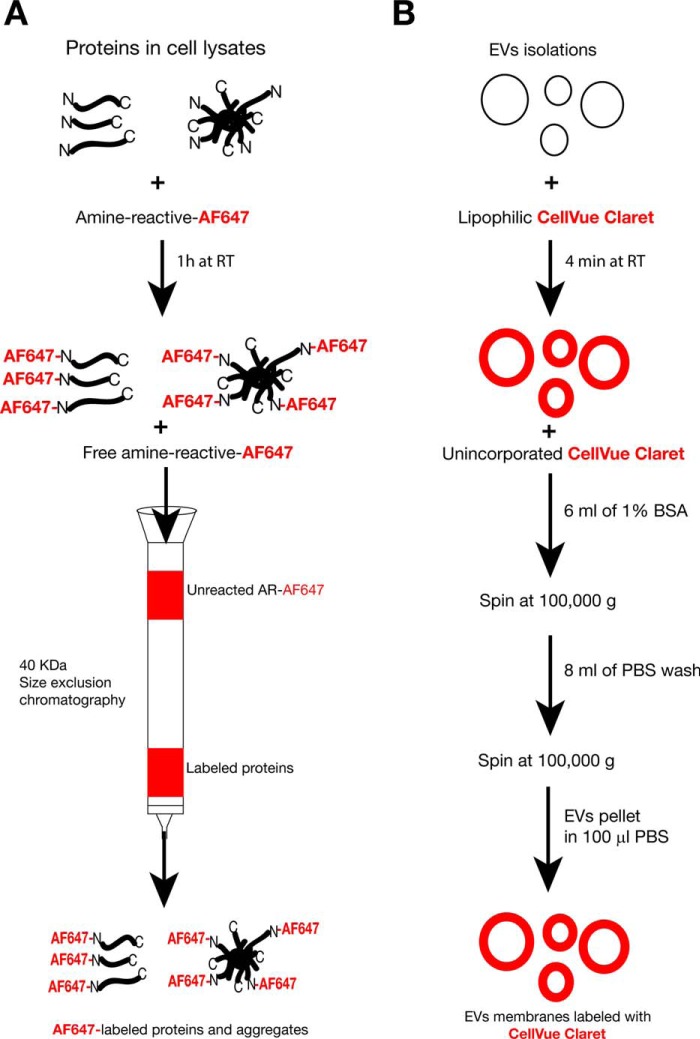
**Far-red fluorescent labeling of proteins and exosome-like EVs.** *A*, the strategy for labeling of free proteins and protein aggregates present in brain cell lysates. Amine-reactive Alexa Fluor 647 (AF647) labels the available N termini. Unreacted label is discarded by size exclusion chromatography, which recovers only AF647-labeled proteins and aggregates above 40 kDa. *RT*, room temperature. *B*, outline of the labeling of EV membranes with a lipophilic far-red dye (Cell Vue Claret). Lipophilic Cell Vue Claret incorporates with high affinity into the lipid bilayer of EV membranes. Traces of unbound dye are chelated with 1% BSA, and labeled exosome-like EVs are further purified by ultracentrifugation and PBS washes. It is important to note that the fluorescent label is in the membranes of exosome-like EVs, not the proteins.

**FIGURE 7.**
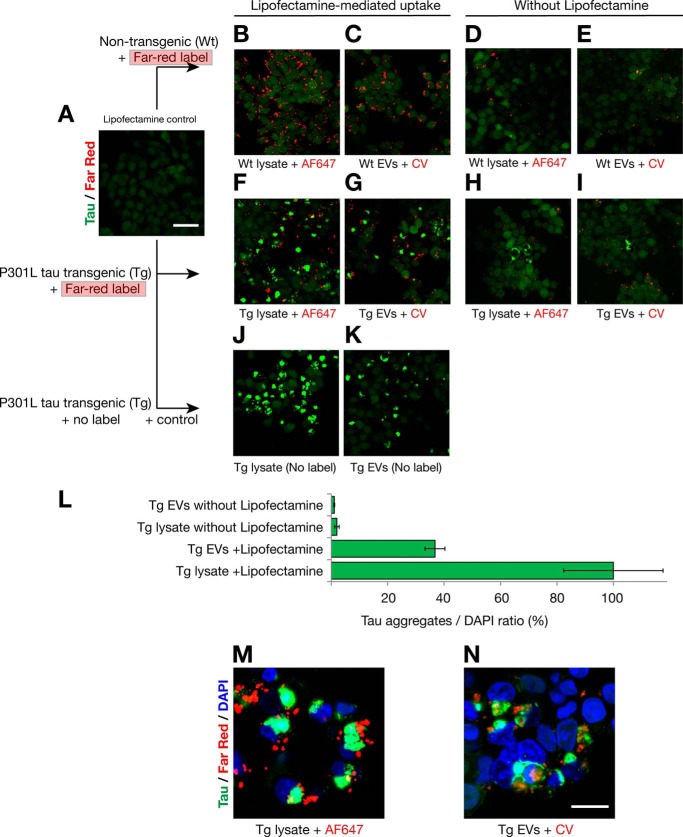
**Lipofectamine increases the uptake of EVs and protein aggregates that trigger intracellular tau inclusions that are closely associated with internalized seeds.** *A–K*, tau biosensor cells were treated in the presence (*B*, *C*, *F*, *G*, *J*, and *K*) or absence (*D*, *E*, *H*, and *I*) of Lipofectamine, analyzed by confocal microscopy after 24-h treatment. To visualize uptake by cells, cell lysates were labeled on the N terminus of proteins with AF647. Membranes of EVs were labeled with the lipophilic far-red dye Cell Vue Claret (CV) for a similar internalization assay. Endogenous tau was detected by green/yellow emission (530–600 nm). *Scale bars* = 50 μm (*A–K*) and 20 μm (*M* and *N*). *A*, FRET tau biosensor cells treated with Lipofectamine vehicle only. No far-red signal is detected because no exosome-like EVs or cell lysates were added. *B–E*, none of the treatments with labeled WT exosome-like EVs or WT cell lysates induces aggregation of endogenous tau. *F–I*, treatments with labeled Tg cell lysates or exosome-like EVs. Bright tau inclusions are visualized with AF647 Tg cell lysates (*F*) and CV-Claret Tg EVs (*G*) in the presence of Lipofectamine. Tau inclusions are also detected with AF647 Tg cell lysates (*H*) or CV-Claret Tg EVs (*I*) in the absence of Lipofectamine. *J–K*, Lipofectamine-mediated uptake of P301L Tg cell lysates and exosome-like EVs without far-red label, used as positive controls. *L*, quantification of the ratio of the areas of induced tau aggregates normalized over the area of nuclei detected 24 h after treatment for Tg samples shown in *F–I* (tau aggregates/DAPI in percent, *n* = 4). *M* and *N*, high-magnification images of the internalization of far-red labeled Tg cell lysates (*M*) and Tg exosomes (*N*) in the presence of Lipofectamine. *M*, tau inclusions form in close proximity to internalized exogenous protein aggregates, sometimes completely surrounding the seed. *N*, this is also observed for internalized exosome-like EVs, where membranes are still showing Cell Vue Claret stain, whereas EVs nucleate the formation of intracellular tau inclusions. The signal of Cell Vue Claret suggests that there is a compartmentalized membrane involved in the presentation of tau seed to the cell for triggering aggregation.

**FIGURE 8.**
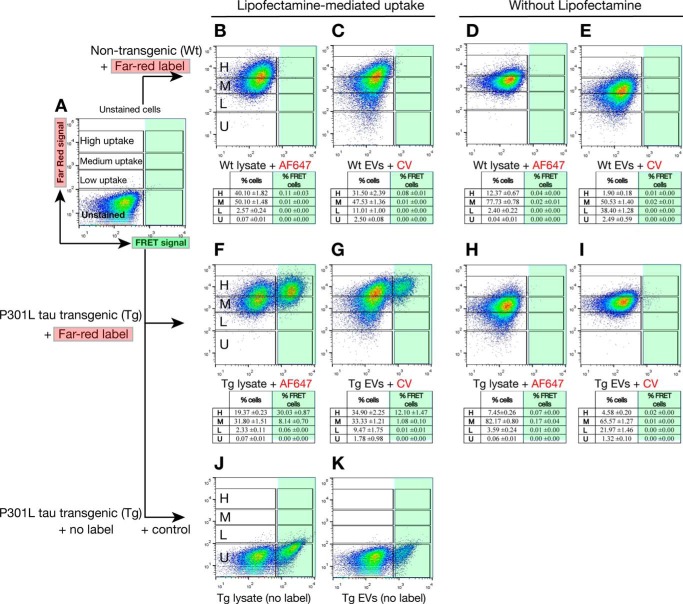
**Levels of uptake of exosome-like EVs and proteins in cell lysates determine the generation of the FRET signal.** Tau biosensor cells were treated in the presence (*B*, *C*, *F*, *G*, *J*, and *K*) or absence (*D*, *E*, *H*, and *I*) of Lipofectamine and analyzed by FRET flow cytometry after a 24-h treatment. To perform quantification of uptake, protein cell lysates were labeled with AF647 and membranes of EVs with CV Claret. Arbitrary gates representing different levels of uptake (low, *L*; medium, *M*; high, *H*; unstained cells, *U*) are shown. The percentage of cells in each of the gates shown is depicted in the corresponding individual table below each flow cytometry plot (*n* = 3, average ± S.D., 40,000 cells/experiment were analyzed). *A*, FRET tau biosensor cells do not show FRET when treated with Lipofectamine vehicle only (right gates, shaded *green*) and show no far-red signal because no exosome-like EVs or cell lysates were added. *B–E*, none of the treatments with labeled WT exosome-like EVs or WT cell lysates induces FRET. However, far-red analysis of the uptake shows that the tau biosensor cells internalized proteins in cell lysates and whole EVs from WT samples in the absence of Lipofectamine (*D* and *E*), but uptake increased with Lipofectamine (*B* and *C*). *F* and *G*, Tau biosensor cells treated with Tg cell lysates or EVs in the presence of Lipofectamine. A strong FRET signal is detected with AF647 Tg cell lysates (*F*) and CV Claret Tg exosome-like EVs (*G*). In the presence of Lipofectamine, the majority of cells reached a high level of uptake. Consequently, the majority of cells showing FRET are those with the highest level of uptake of far-red labeled Tg sample. *H* and *I*, no FRET events are detected with AF647 Tg cell lysates (*H*) or CV Claret Tg EVs (*I*) in the absence of Lipofectamine. The majority of cells only reached a medium level of uptake. *J* and *K*, treatments with P301L Tg cell lysates and exosome-like EVs without the far-red label were used as a positive control.

To address these three possibilities, we fluorescently labeled the membranes of exosome-like EVs with Cell Vue Claret (a lipophilic far-red dye) and the amino terminus of proteins and aggregates in cell lysates with an amine-reactive Alexa Fluor 647 (Fig. 6). We added 20 μg of protein equivalents of far-red-labeled EVs and cell lysates to visualize the uptake of the different samples by the tau biosensor cells using confocal microscopy (Fig. 7) and to quantify the efficiency and levels of uptake using FRET flow cytometry (Fig. 8).

Again, a vehicle control was included (Fig. 7*A*), testing wild-type (Fig. 7, *B–E*) and transgenic samples (Fig. 7, *F–N*). Confocal analysis showed that intracellular inclusions of endogenous tau were robustly visualized only when the sample, either exosome-like EVs or cell lysate, was isolated from the tau transgenic mice (Fig. 7, *F–I*). However, the induced tau inclusions were much bigger and highly frequent in the presence of Lipofectamine (Fig. 7, *F* and *G*). However, even without Lipofectamine, although they were rare, tau inclusions were present. Their size was reduced using, as exogenous seeds, either transgenic cell lysates (Fig. 7*H*) or transgenic EVs (Fig. 7*I*), supporting scenario 1, *i.e.* the presence of a threshold for the induction of endogenous tau inclusions. Quantification of tau aggregates demonstrates that the formation of tau inclusions after only 24 h when no Lipofectamine is used is a rare event (Fig. 7*L*). Interestingly, high-magnification images showed that tau inclusions formed in close proximity to internalized exogenous seeds (Fig. 7, *M* and *N*), surrounding the proteinaceous seed (Fig. 7*M*) or the EVs (Fig. 7*N*). This indicates an active role of exogenous seeds in the nucleation of the nascent intracellular tau inclusions. However, in the case of exosome-like EVs, the presence of the Cell Vue Claret signal indicates that there are membranes mediating the presentation of the tau seed contained in EVs and that these membranes do not impair the presentation and induction of tau aggregation (Fig. 7*N*).

##### Vesicle Uptake in the Absence of Lipofectamine: Only Cells with a High Degree of Exosome-like EV Uptake Generate FRET Events of Tau Aggregation

Our confocal microscopy data support the notion that high levels of uptake are required to strongly induce the aggregation of endogenous tau in recipient cells (Fig. 7). We therefore attempted to quantify, by FRET flow cytometry, how different levels of uptake contribute to the generation of FRET events in tau biosensor cells. To achieve this, we used the same exosome-like EVs labeled with Cell Vue Claret and cell lysates labeled with Alexa Fluor 647 to track their internalization by tau biosensor cells. We then established arbitrary cytometry gates for different levels of uptake (low (L), medium (M), and high (H) compared with the unstained control (U)) and analyzed these uptake levels against their ability to induce FRET events of tau aggregation (Fig. 8).

As above, a vehicle control was included (Fig. 8*A*), and wild-type (Fig. 8, *B–E*) and tau transgenic samples were tested (Fig. 8, *F–K*). FRET flow cytometry showed that EVs labeled with Cell Vue Claret were robustly taken up by FRET tau biosensor cells even without using Lipofectamine (Fig. 8, *E* and *I*). Similarly, Alexa Fluor 647-labeled lysates were taken up by cells in the absence of Lipofectamine (Fig. 8, *D* and *H*). However, using Lipofectamine strongly increased the uptake of both EVs (Fig. 8, *C* and *G*) and cell lysates (Fig. 8, *B* and *F*). For instance, without Lipofectamine, only 1.9% of cells show a high level of EV uptake (Fig. 8*E*), whereas, with Lipofectamine, 31.5% of cells internalize high levels of exosome-like EVs (Fig. 8*C*). When the FRET signal was analyzed in parallel, we observed that the majority of cells showing FRET were those exhibiting the highest level of uptake for both transgenic EVs (Fig. 8*G*) and cell lysates (Fig. 8*F*). In the absence of the far-red label, as in the positive control of the experiment, all FRET events were detected opposite to unstained gates (Fig. 8, *J* and *K*). These results support the presence of a threshold beyond which endogenous tau aggregation can be induced (Fig 10), with implications for the spreading of tau pathology. Although we have shown that Lipofectamine is needed to detect the induction of tau aggregation by FRET flow cytometry at 24 h post-treatment (Fig. 4, *I* and *J*), longer incubation proved that both transgenic EVs and transgenic cell lysates can induce tau aggregates in the absence of Lipofectamine by FRET flow cytometry (Fig. 5, *H* and *I*) and also by confocal microscopy analysis (Fig. 5, *Q–S*). These aggregates are more pronounced when a FRET signal is present, together underscoring the presence of a threshold in tau aggregate induction by exosome-like EVs.

##### Alix and Flotillin-1 Immunocolocalized in Isolated EVs and Internalized in Cells

**FIGURE 9.**
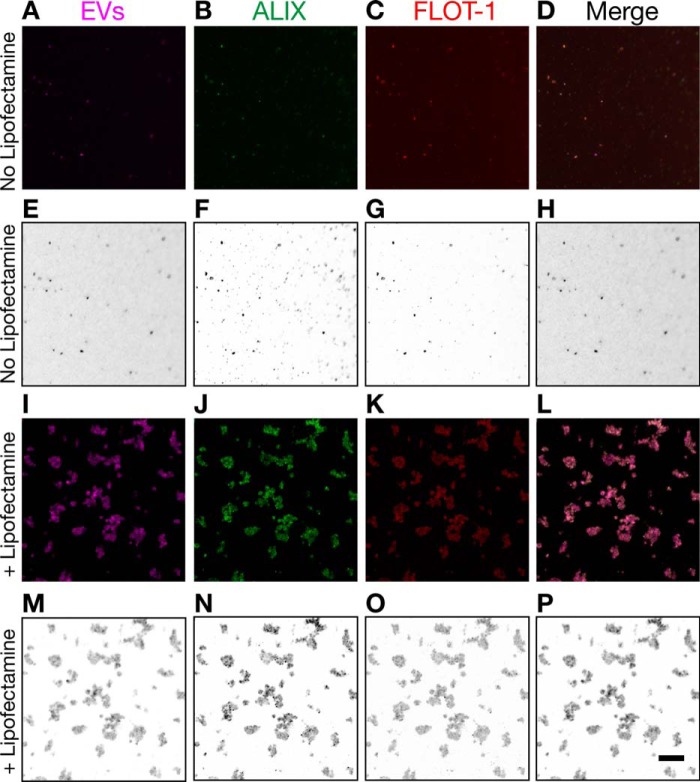
**Induction of EV clusters and immunocolocalization of Alix and flotillin-1.** *I–L*, mouse brain-derived EVs labeled with Cell Vue Claret and treated with Lipofectamine for immunofluorescence analysis of Alix and flotillin-1. *A–D*, EVs not treated with Lipofectamine were used as a control. Corresponding grayscale-inverted look-up table images are shown below each immunofluorescence image for better visualization (*E–H* and *M–P*). Shown is colocalization of Alix, expressed mostly in a punctuated fashion (*bright green dots*), and flotillin-1. Colocalization occurs independent of Lipofectamine treatment. However, Lipofectamine increases the binding capacity of EVs and induces the formation of EV clusters, which also increased the sensitivity of the immunofluorescence detection. Clusters of EVs varied in size. *Scale bar* = 10 μm.

Given that Lipofectamine increased the uptake of EVs (Fig. 8), we tested whether Lipofectamine could increase the adherence capacity of isolated EVs as a potential mechanism to increase their cellular uptake. At the same time, we wanted to investigate whether Alix and flotillin-1 colocalized in exosome-like EVs isolated from mouse brains. Therefore, we treated exosome-like EVs labeled with Cell Vue Claret with Lipofectamine as if they were going to be used for cellular uptake. As a control, we used EVs without Lipofectamine. Then we placed the EVs on poly-d-lysine-coated coverslips in a similar way as done before for cellular uptake. After an overnight incubation, we directly fixed the EVs without washing the coverslips and performed immunofluorescence for Alix and flotillin-1. Strikingly, treatment with Lipofectamine not only increased the binding to coverslips but strongly increased the binding between EVs, inducing the formation of EV clusters of varied size (Fig. 9, *I–P*). This indicated that clusters of EVs taken up by cells may increase the number of intracellular tau seeds that can engage in intracellular tau aggregation in recipient cells, thus explaining the strong effect of Lipofectamine in the seeding of tau biosensor cells. The formation of EV clusters also increased the visualization by immunofluorescence, revealing robust colocalization of Alix and flotillin-1 (Fig. 9, *J–M*). A similar colocalization was obtained for the EVs that had not been incubated with Lipofectamine, although the fluorescence signal was weaker because of the lower number of EVs bound to the coverslips (Fig. 9, *A–D*).

**FIGURE 10.**
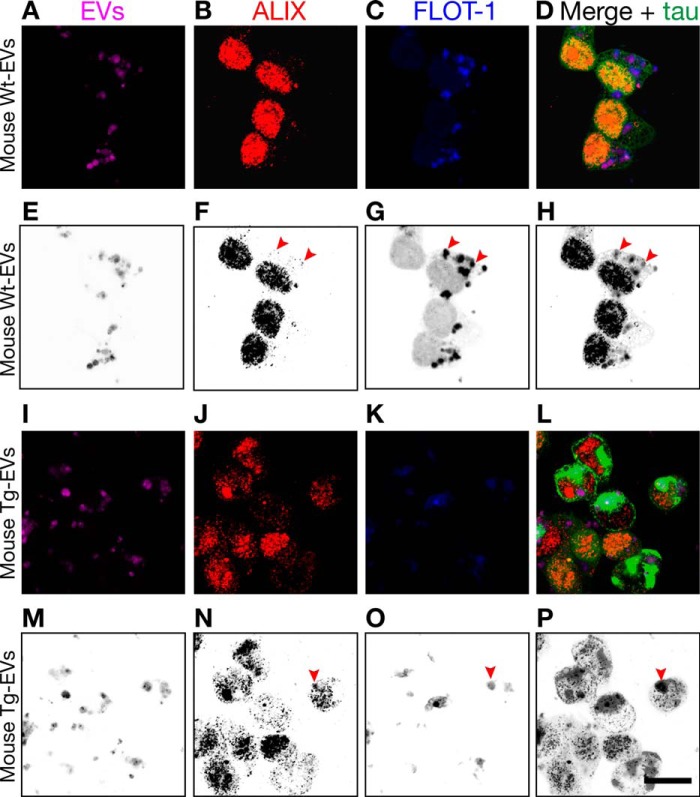
**Alix and flotillin-1 are also immunocolocalized on EVs after cellular uptake.** Shown is Lipofectamine-mediated internalization of mouse brain-derived EVs labeled with Cell Vue Claret by tau biosensor cells. The *arrowheads* indicate sites of Alix and flotillin-1 colocalization. The corresponding grayscale-inverted look-up table images are shown below each immunofluorescence image for better visualization (*E–H* and *M–P*). *A–D*, exosome-like EVs from WT mice (*Wt-EVs*) were robustly internalized but failed to engage in tau aggregation. However, ALIX, forming intracellular puncta, and FLOT-1 colocalized in internalized EVs (*arrowheads*). *I–L*, exosome-like EVs from rTg4510 transgenic mice (*Tg-EVs*) strongly induced intracellular tau inclusions (*bright green*) in recipient cells. In the middle of this reorganized cytoplasm, some colocalization of ALIX and FLOT-1 is observed, sometimes buried in EVs inside tau inclusions (*arrowheads*). *Scale bar* = 20 μm.

Next, we aimed to investigate Alix and flotillin-1 colocalization after cellular uptake. As expected, we found that Alix and flotillin-1 colocalized on exosome-like EVs labeled with Cell Vue Claret after internalization by the tau biosensor cells (Fig 10).

##### Dose-dependent Effect of Lipofectamine in the Induction of FRET Events of Tau Aggregation

**FIGURE 11.**
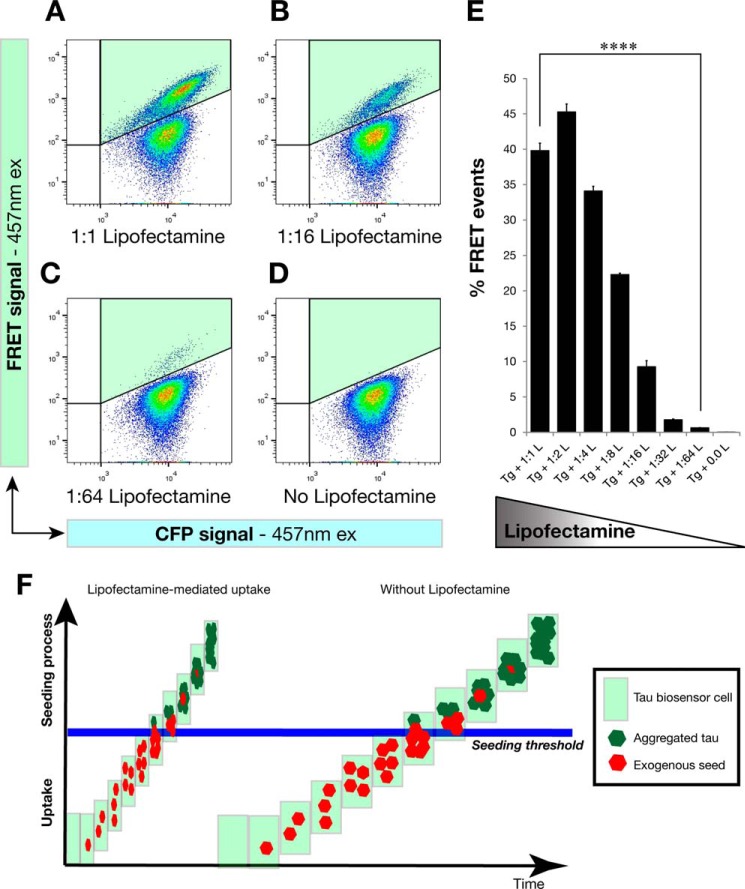
**Lipofectamine increases the uptake of exogenous seeds and accelerates the seeding process in a dose-dependent manner.** Tau biosensor cells were treated with the same amount of transgenic cell lysate for all conditions shown. However, Lipofectamine was gradually decreased at 2-fold dilutions and analyzed by FRET flow cytometry after a 24-h treatment (*n* = 3, average ± S.E., 40,000 cells analyzed per experiment). *A–D*, flow cytometry plots of representative treatments showing that the less Lipofectamine was added to the treatment the less FRET events of tau aggregation were induced. The FRET signal is detected in the right gate (Q2, shaded in *green*). *ex*, excitation. *E*, quantification of the percentage of FRET events detected with the different treatments, revealing a dose-dependent response to Lipofectamine concentrations. *Error bars* represent S.E. (*n* = 3). ****, *p* < 0.0001. *F*, concept of the action of Lipofectamine on treatments of tau biosensor cells. Our study supports the notion that Lipofectamine increases the uptake of exogenous tau seeds, which has a profound effect on the cellular decision of forming tau inclusions. High levels of uptake of misfolded tau seeds are required to trigger endogenous tau seeding inside the cell. This is compatible with the operation of a seeding threshold involved in this cellular decision. In the absence of Lipofectamine, the seeding process is slow and inefficient, which might be one of the reasons why tau pathology progresses very slowly in human patients with tauopathy.

Having demonstrated that high levels of exogenous seed uptake are necessary for endogenous tau aggregation and that Lipofectamine increases such an uptake, probably by increasing the number of internalized tau seeds in the form of EV clusters, we set out to investigate whether the effect of Lipofectamine occurs in a dose-dependent manner. We thus treated FRET tau biosensor cells with the same amount of transgenic cell lysate and gradually decreased, in 2-fold dilutions, the amount of Lipofectamine that was mixed with the transgenic lysates (Fig 11, *A–D*). We quantified the results by FRET flow cytometry after a 24-h treatment (Fig 11*E*) and observed that indeed there was a dose-dependent effect of the concentration of Lipofectamine on the percentage of FRET events that were detected. Therefore, keeping the exogenous tau seeds constant and only changing the amount of Lipofectamine resulted in a dose response of FRET events of tau aggregation (Fig 11*E*). Our concept of the action of Lipofectamine on treatments of tau biosensor cells is shown in Fig. 9*F*, where Lipofectamine increases the uptake of exogenous seeds and accelerates the seeding process.

##### Only Cells Harboring Tau Inclusions Secrete EVs Carrying Tau Seeds

**FIGURE 12.**
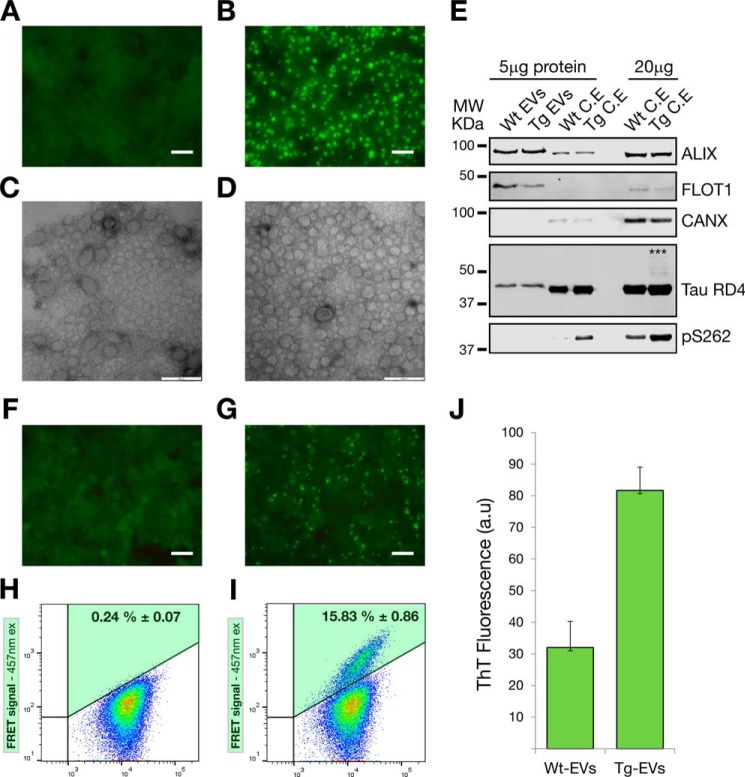
**Only cells harboring tau inclusions secrete exosome-like EVs carrying tau seeds.** Tau biosensor cells were used as an *in vitro* cellular model to isolate exosomes from a cell line in which tau aggregation was induced. *Scale bar* = 50 μm (*A*, *B*, *F*, and *G*) and 100 nm (*C* and *D*). *A*, fluorescent live-cell imagining of tau biosensor cells treated with WT mouse brain cell lysates to generate cultures without tau aggregation. *B*, cultures of cells forming tau inclusions (*bright green aggregates*) were obtained by treating the tau biosensor cells with rTg4510 brain lysates. *C* and *D*, electron microscopy of exosome-like EVs isolated from CCM of WT (*C*, *Wt-EVs*) or Tg cell cultures (*D*, *Tg-EVs*), showing that both types of cultures generate mostly EVs of less than 100 nm in diameter. *E*, Western blotting analysis of isolated EVs. 5 μg of protein equivalents of sucrose-purified EVs (F3, 0.95 m sucrose) were blotted. 5 and 20 μg of protein of corresponding cell lysates are included for comparison. Using equivalent protein quantities, both EVs and cell lysates show strong up-regulation of the exosome markers ALIX and FLOT-1. The endoplasmic reticulum marker calnexin (*CANX*) was undetectable. Total tau was detected with the Tau-RD4 antibody, showing that tau is present at similar levels in both WT and Tg EVs. SDS-stable high molecular weight (*MW*, *asterisks*) tau species were only detected with 20 μg of transgenic cell lysates. Increased tau phosphorylation (Ser(P)-262) was detected in cells undergoing cellular tau aggregation. However, Ser(P)-262 was undetectable with the amount of EV protein analyzed. *C.E*, cell extract. *F*, exosome-like EVs from WT cultures (WT EVs + Lipofectamine) are unable to induce tau aggregation in naïve tau biosensor cells. *G*, fluorescent live-cell imagining of tau biosensor cells treated with Tg EVs + Lipofectamine, showing the robust presence of induced tau aggregates. *H* and *I*, FRET flow cytometry of treatments with 5 μg of protein equivalents of WT EVs (*H*) and Tg EVs (*I*) showing that only Tg EVs are able to generate FRET signals indicative of tau aggregation (*n* = 3, average ± S.D., 40,000 cells analyzed per experiment). *ex*, excitation. *J*, ThT fluorescence assay of HEK293-derived EVs (*n* = 3, average fluorescence ± S.E.; *a.u.*, arbitrary units). A comparatively stronger increase of ThT fluorescence in Tg EVs indicates a higher presence of misfolded and aggregated tau.

Our findings with exosome-like EVs isolated from mouse brains support the notion that only cells undergoing pathological aggregation of tau are able to generate EVs carrying tau seeds. We decided to test this hypothesis by isolating exosomes prepared from a cell line in which tau aggregation was induced. For cells without tau aggregation, we used tau biosensor cells treated with WT mouse brain cell lysates (Fig 12*A*) intended to generate WT EVs. Also, cells forming tau inclusions were obtained by treating the tau biosensor cells with transgenic brain lysates (Fig 12*B*), a potential source of Tg EVs. CCM from these two cell cultures were used to isolate exosome-like EVs (WT EVs and Tg EVs) by centrifugation using standard methods (34), followed by further purification on a sucrose gradient, as done before to prepare mouse brain EVs. We corroborated that we had exosomes in sucrose F3 (0.95 m sucrose), as determined by electron microscopy (Fig 12, *C* and *D*) and Western blotting analysis, which showed a strong presence of the exosome markers Alix and flotillin-1 (Fig 12*E*). Calnexin was undetectable in isolated EVs (Fig 12*E*), supporting the notion that the preparations were pure. The tau RD expressed in the tau biosensor cells only comprises amino acids 244–372 compared with 441 amino acids for the longest human tau isoform (35). Therefore, total tau is only detectable with the tau-RD4 antibody, which showed that tau is present at similar levels in both WT and Tg EVs, indicating that the intracellular tau aggregation did not affect the packaging of tau into exosomes (Fig 12*E*). To detect an increase of tau phosphorylation, we used the Ser(P)-262 antibody that targets an internal epitope in the short tau RD. However, although we failed to detect any phosphorylation in the EVs, we observed an increase in phosphorylation in the corresponding Tg cell lysates (Fig 12*E*). We assumed that Ser(P)-262 is undetectable in EVs because of the comparatively lower presence of tau in EVs, in conjunction with the low percentage of total tau that is actually phosphorylated. Therefore, it is likely that, with much more protein being present, such a phosphorylation could be detected. A role for protein content in detecting a signal was also evident when tau-RD4 was used for detection. Only with 20 μg of Tg cell lysate was it possible to visualize high molecular weight tau species that formed in cells having tau inclusions, which are SDS-stable and reduction-resistant (Fig 12*E*).

Intriguingly, despite showing a similarity in the content of total tau (Fig. 12*E*), when WT and Tg EVs were assayed on naïve tau biosensor cells, only Tg EVs were able to induce the formation of tau inclusions (Fig 12*G*). This was further confirmed and quantified by FRET flow cytometry, which showed FRET events of tau aggregation only in cells treated with Tg EVs (Fig 12*I*). All of these functional data support the presence of tau seeds only in Tg EVs. We reasoned that tau seeds in Tg EVs should be misfolded conformers of tau and that therefore the fluorescent dye ThT would reveal the increased presence of aggregated tau specifically in Tg EVs. The enhanced ThT fluorescence supports the notion that Tg EVs indeed carry higher quantities of misfolded and aggregated tau than WT EVs (Fig 12*J*).

## Discussion

Protein aggregation is a defining feature of neurodegenerative disease. Aβ and tau form aggregates in AD and related tauopathies, as does α-synuclein in Parkinson disease and TDP-43 and FUS in amyotrophic lateral sclerosis (36). A commonality of these proteins is their susceptibility not only to aggregate but also to form seeds with characteristics similar to prions, including the molecular properties of nucleation, templating, growth, multiplication, and spreading through the brain (36). However, the actual nature of the seeds and the mechanism by which they escape diseased cells and are transmitted to healthy neurons to induce aggregation has not been established in detail. Here we show that P301L tau-containing exosome-like EVs carry tau seeds that are capable of inducing aggregation of endogenous tau after being taken up by recipient tau biosensor cells, confirming that EVs present a vehicle to transmit tau pathology.

In this study, we first demonstrated that exosome-like EVs can be isolated from the extracellular space of the mouse brain and that they contain significant amounts of tau. To our knowledge, a strong association between exosomes and phosphorylated tau has been demonstrated in AD patients (18, 21) but not in cultured neurons (27, 30). Therefore, we reasoned that, to isolate neuron-derived exosome-like EVs carrying phosphorylated tau and potential tau seeds, we needed to isolate them from neuronal tissue with a tau pathology. Indeed, we identified tau in exosome-like EVs isolated from rTg4510 mice. However, this tau was only weakly phosphorylated, and some critical and pathological phosphorylation-dependent epitopes, such as AT8, AT100, and AT180, were undetectable. We cannot claim that these epitopes are not present, but their expression was much lower than in brain extracts, rendering them undetectable in EVs by Western blotting. In contrast, phospho-epitopes such as Ser(P)-262, Ser(P)-422, and AT270 were present, and AT270 is a phospho-epitope that has been strongly detected in AD-derived human exosomes (21). AT270 was also the epitope that in our study presented with one of the strongest signals in exosome-like EVs isolated from tau transgenic mice. Given that pathological epitopes such as AT8, AT100, and AT180 have been found to be associated with pathological forms of tau (37, 38), this suggested to us that the cargo in exosome-like EVs might not contain large amounts of aggregated tau. In agreement with this, only by overexposing the total tau blots did we find evidence for aggregated tau in exosome-like EVs. The high molecular weight tau species showed a signal that in size is compatible with tau trimers, the minimal tau propagation unit that is able to induce endogenous tau aggregation (39). This type of SDS-stable and reduction-resistant aggregated form of tau has been detected in AD patients (40–42) and also in transgenic mouse models of tau pathology (43). Interestingly, SDS stability of *in vitro* aggregated tau can only be achieved by chemical cross-linking (42). In our study, the high molecular weight tau species showed the strongest signal in F3 that contains the majority of exosomes. Although this has not been investigated, it is tempting to speculate that perhaps some form of size exclusion mechanism may be in place that only allows oligomers of tau to be packaged into exosomes and that some tau phospho-species are more compatible with such packaging than others.

A major aim of our study was to determine whether exosome-like EVs carry tau in a form that could act as a seed of endogenous tau aggregation in recipient cells. To make this possible, we expected that the exosomal tau had to be aggregated because monomeric tau clearly lacks seeding activity (5–7, 44). We also inferred that tau had to be phosphorylated because *in vivo* tau aggregates are usually hyperphosphorylated (45), and tau phosphorylated at AD sites polymerizes more readily into filaments (46). Our Western blotting analysis with brain-derived EVs supports the notion that such expectations could be met with transgenic exosome-like EVs and that we could proceed with functional assays. However, conformation and the capacity to form a β sheet structure are the main features behind the seeding ability of aggregated tau (5, 35, 44, 47, 48). Interestingly, although unphosphorylated and *in vitro* aggregated tau can seed tau aggregation, *in vivo* derived aggregated tau is more efficient in seeding the aggregation in recipient cells than tau that has been aggregated and even phosphorylated *in vitro* (44). Our data show that, although relative tau levels (compared with total protein contents) in mouse exosome-like EVs were ∼3-fold lower than in cell lysates, and tau was also less phosphorylated, the tau seeds present in transgenic exosome-like EVs were sufficient to induce tau aggregation and inclusions in recipient tau biosensor cells. Nevertheless, the ability to induce FRET events was clearly much lower than for cell lysates, which also demonstrates that the differential contents of tau and/or phosphorylated tau could underpin the comparatively lower efficiency of exosome-like EVs. However, by using a complete cell lysate, this is the equivalent of a cell releasing its entire cellular contents into the extracellular space. *In vivo*, this occurs only after neuronal death, at an advanced stage of AD (49). However, EV-mediated tau spreading quite likely happens while neurons undergo tau aggregation but are still alive. In such a situation, secretion of extracellular vesicles and even the secretion of membrane-free tau could be the predominant mechanism in the spreading of tau pathology before neurodegeneration occurs, boosting tau pathology.

Furthermore, given that increased tau phosphorylation is associated with tau aggregation *in vivo* (45, 46, 50), carrying phosphorylated tau indicated the likely presence of tau seeds with the ability to induce intracellular tau aggregation. Therefore, WT samples containing lower levels of tau that are less phosphorylated were not able to induce FRET events, even when much higher concentrations of WT cell lysates were used.

Interestingly, both extracellular vesicles and protein aggregates are taken up by recipient cells using the endocytic pathway (5, 51–54), and quite likely such a mechanism was operational in our experiments with the tau biosensor cells. Our experiments with EVs and protein lysates revealed an enhanced internalization of both exosomes and protein aggregates in the presence of Lipofectamine, indicating the existence of an activation threshold that controls intracellular aggregation. This threshold was lowered in the presence of Lipofectamine. The mode of action of Lipofectamine appears to be by increasing the binding capacity of EVs and inducing the formation of EV clusters. Endocytosis of EV clusters instead of individual EVs quite likely increases the number of intracellular tau seeds that are available to trigger intracellular tau aggregation in recipient cells.

We found that the power of FRET flow cytometry to predict the presence of tau inclusions in the absence of Lipofectamine was lower than that of confocal microscopy at 24-h experiments. We consider that factors such as the smaller size of the tau aggregates formed without Lipofectamine and the low numbers of these events could have contributed to the apparently lower sensitivity of flow cytometry. However, when a longer, 72-h incubation post-treatment was allowed, tau aggregation in the absence of Lipofectamine became detectable using FRET flow cytometry. Apart from longer incubation, it is likely that the lower number of cells used for the 72-h experiments could have increased the molar concentration of tau available per cell, thereby increasing the probability of reaching the induction threshold of tau aggregation for some cells. Nevertheless, FRET flow cytometry was sensitive enough to demonstrate that another critical factor in the formation of intracellular aggregates is time because tau transgenic cell lysates and exosome-like EVs were able to induce endogenous tau aggregation in the absence of Lipofectamine when more time was allowed.

Our data obtained with brain-derived exosome-like EVs isolated from rTg4510 mice support the notion that only cells undergoing pathological aggregation of tau are able to secrete EVs carrying tau seeds. WT and Tg EVs from HEK293 cells exhibited similar levels of tau by Western blotting, but when assessed with naïve tau biosensor cells, only the exosomes coming from cells undergoing tau aggregation were able to trigger the formation of tau inclusions in recipient cells. Therefore, the conformation of tau in Tg EVs and not the differences in its concentration is the driving force triggering tau aggregation. This notion was corroborated by enhanced ThT fluorescence in Tg EVs, indicating the higher presence of misfolded and aggregated tau. These *in vitro* results are in agreement with our data obtained with EVs isolated from rTg4510 mouse brains, where phosphorylated and oligomeric tau seem to be the active conformers in the tau seeds.

Concomitantly, our tau phosphorylation analysis suggested that mouse brain exosome-like EVs are likely carrying limited amounts of aggregated tau, as also supported by the low amount of high molecular weight tau species detected by Western blotting. In comparison, in tau biosensor cells, the engineered tau RD does not contain sites for phosphorylation of pathological tau (*i.e.* AT8, AT100, AT180, AT270, and Ser(P)-422). However, sites still susceptible to phosphorylation, such as Ser(P)-262, were increased in phosphorylation when tau RD was aggregated in the cells. Nevertheless, HEK293 Tg EVs were able to induce tau aggregation in naïve recipient cells despite not showing these other pathological phosphorylation events. This may indicate that tau phosphorylation works mostly by detaching tau from microtubules rather than influencing the seeding capabilities of tau.

However, tau seed levels in EVs are sufficient to induce misfolding and aggregation of tau in recipient cells provided that concentrations are above the activation threshold. Therefore, the small amount of aggregated tau in exosome-like EVs could result in a slow seeding in neurons, which could be one factor contributing to the slow progression of AD. Nevertheless, it has been reported recently that microglia promote tau propagation by phagocytosing tau-containing cytophagic neurons or synapses and subsequently secrete tau in exosomes (24). Therefore, tau-containing extracellular vesicles derived from microglia quite likely contributed to the extracellular pool of tau we analyzed, underpinning the spreading and progression of tau pathology.

Also, it is tempting to speculate that the presence of an activation threshold in the decision of triggering tau aggregation has significant effects in the development of tau pathology. Our data clearly show that internalization occurs naturally without the assistance of lipofactors but that the formation of tau inclusions required higher levels of internalized seeds to trigger the process. This implies that reducing the pool of extracellular tau seeds, irrespective of whether these are moving freely or are transported by tunneling nanotubes or EVs, could result in a reduction of tau pathology by keeping tau seeds below a threshold concentration.

It has been debated whether transmissible protein aggregates such as tau are truly “prions,” defined as “proteinaceous infectious particles,” given that protein aggregates that seed neurodegenerative lesions are not infectious by any conventional definition (2, 55, 56). This situation has encouraged the redefinition of prions as “proteinaceous nucleating particles” to highlight the molecular action of the agents of neurodegenerative noninfectious proteinopathies (2). This novel definition fits with our observation that exogenous tau seeds, in the form of either free aggregates or exosome-like EVs, were found in close proximity to endogenous intracellular tau inclusions. We also found evidence that some of those seeds were not only close to but also completely surrounded and covered by the tau inclusion, sometimes masking the far-red signal from the seed. This supports an active role of the exogenous seed in nucleating nascent tau inclusion but also suggests that, to maintain neuron-to-neuron spreading, *de novo* tau seeds need to be generated because those that are initially formed appear to become trapped by the tau inclusions.

We also observed that the far-red signal used to label EV membranes was still present during the nucleation and formation of tau inclusions. This suggests the presence of membranes mediating the presentation of the tau seed that is contained in EVs. Alternatively, they could be highlighting a membrane-bound subcellular compartment from which EV membranes cannot get out or are degraded. However, at the level of resolution of our confocal microscopy analysis, it is difficult to determine whether the EVs are intact or are disrupted EV membranes. Furthermore, based on EV size, we are quite likely observing clusters of EVs. Because we and others (22, 24) have found tau inside EVs, it is likely that EV membranes are disrupted inside the cells to expose the internal tau seeds. Moreover, endocytic vacuoles were only clearly observed with labeled mouse WT EVs, which did not trigger tau aggregation. A remaining question is whether such endocytic vacuoles persist when rTg4510-derived EVs trigger intracellular tau aggregation or whether the vacuole membrane is disrupted to expose the tau seeds to the cytoplasm.

It is reasonable to assume that cellular processes targeting the degradation of the internalized tau seeds counterbalance the effects of the seeds. The opposing forces of these processes could control the development and severity of tau pathology *in vivo*. For instance, a growing body of evidence connects decreased autolysosome function to neurodegenerative diseases such as AD (57, 58). It is becoming increasingly clear that multiple factors can converge on the impairment of autolysosomal proteolytic functions. Our results show that overburdening the system with tau aggregates can tip the balance toward the formation of intracellular tau inclusions.

In conclusion, we provide evidence that tau-containing exosome-like EVs can, in a threshold-dependent manner, seed the misfolding and aggregation of endogenous cellular tau. Our results, in conjunction with loss-of-function assays reducing exosome secretion during tau propagation (24), support the strong role of extracellular vesicles in the spreading of tau pathology, unveiling potential pharmacological interventions for AD and related tauopathies.

## Author Contributions

J. C. P. conceived and designed the experiments, collected and/or assembled the data, performed data analysis and interpretation, and wrote the manuscript. B. J. S. conceived and designed the experiments and collected and/or assembled the data. A. F. H. performed data analysis and interpretation and wrote the manuscript. J. G. conceived and designed the experiments, performed data analysis and interpretation, wrote the manuscript, provided financial support, and approved the final version of the manuscript.

## Footnotes

2 The abbreviations used are:

AD: Alzheimer disease
Aβ: amyloid β
EV: extracellular vesicle
APP: amyloid precursor protein
RD: repeat domain
CFP: cyan fluorescent protein
Tg: transgenic
CCM: cell conditioned medium/media
F3: fraction 3
PK: proteinase K
ThT: thioflavin T
ANOVA: analysis of variance
CV: Cell Vue
AF: Alexa Fluor.
